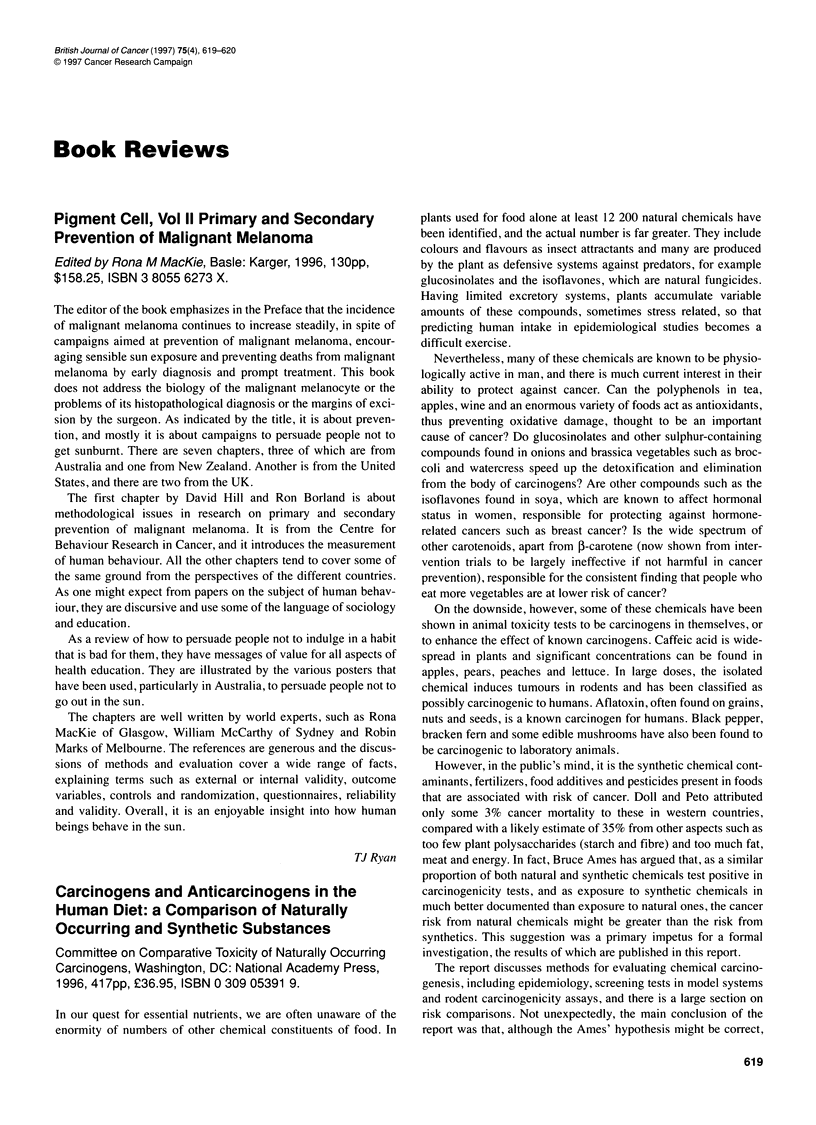# Pigment Cell, Vol II Primary and Secondary Prevention of Malignant Melanoma

**Published:** 1997

**Authors:** TJ Ryan


British Journal of Cancer (1997) 75(4), 619–620
© 1997 Cancer Research Campaign

### Book Reviews

### Pigment Cell, Vol II Primary and Secondary Prevention of Malignant Melanoma

*Edited by Rona M MacKie*, Basle: Karger, 1996, 130pp, $158.25, ISBN 3 8055 6273 X.

The editor of the book emphasizes in the Preface that the incidence of malignant melanoma continues to increase steadily, in spite of campaigns aimed at prevention of malignant melanoma, encouraging sensible sun exposure and preventing deaths from malignant melanoma by early diagnosis and prompt treatment. This book does not address the biology of the malignant melanocyte or the problems of its histopathological diagnosis or the margins of excision by the surgeon. As indicated by the title, it is about prevention, and mostly it is about campaigns to persuade people not to get sunburnt. There are seven chapters, three of which are from Australia and one from New Zealand. Another is from the United States, and there are two from the UK.

The first chapter by David Hill and Ron Borland is about methodological issues in research on primary and secondary prevention of malignant melanoma. It is from the Centre for Behaviour Research in Cancer, and it introduces the measurement of human behaviour. All the other chapters tend to cover some of the same ground from the perspectives of the different countries. As one might expect from papers on the subject of human behaviour, they are discursive and use some of the language of sociology and education.

As a review of how to persuade people not to indulge in a habit that is bad for them, they have messages of value for all aspects of health education. They are illustrated by the various posters that have been used, particularly in Australia, to persuade people not to go out in the sun.

The chapters are well written by world experts, such as Rona MacKie of Glasgow, William McCarthy of Sydney and Robin Marks of Melbourne. The references are generous and the discussions of methods and evaluation cover a wide range of facts, explaining terms such as external or internal validity, outcome variables, controls and randomization, questionnaires, reliability and validity. Overall, it is an enjoyable insight into how human beings behave in the sun.

*TJ Ryan*